# Supplementary figures and images for: SNP-ChIP: a versatile and tag-free method to quantify changes in protein binding across the genome
Source: BMC Genomics. 2019 Jan 17;20:54. doi: 10.1186/s12864-018-5368-4 (PMC6337847; doi:10.1186/s12864-018-5368-4)

**a**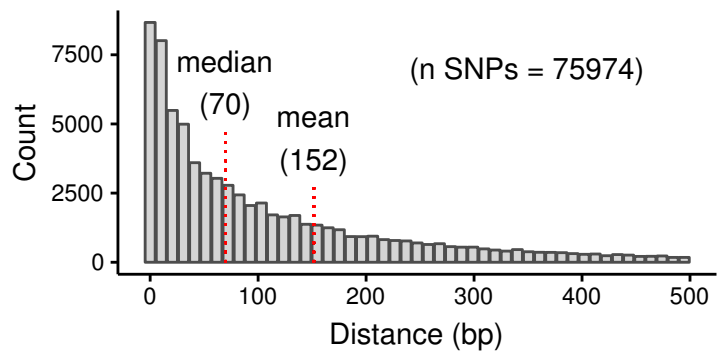**b**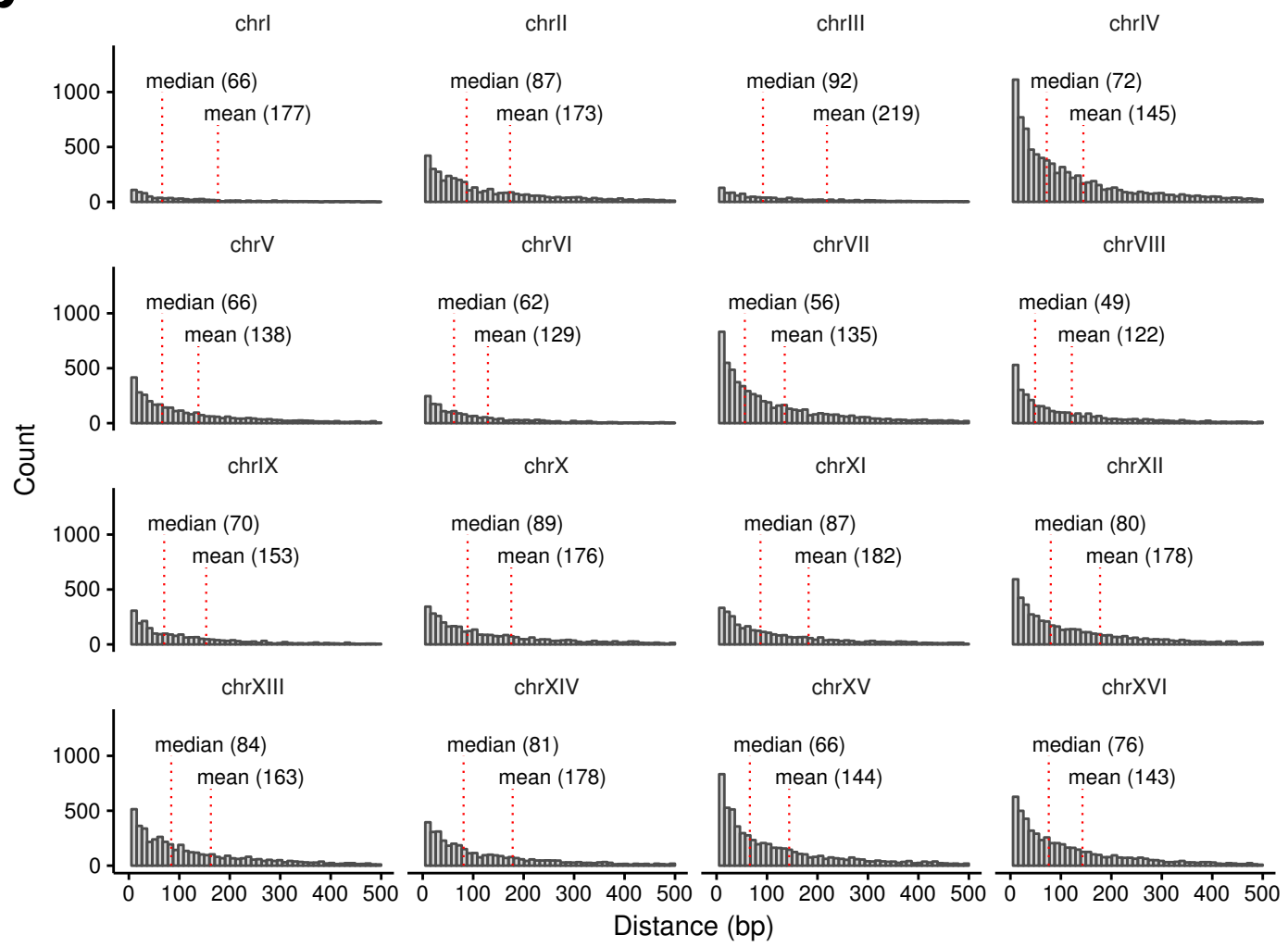

Supplement: Supplementary file 1 — Figure S1. Distribution of distances between consecutive single-nucleotide polymorphisms (SNPs) found between the SK1 and S288c yeast genomes. (PDF 47 kb) [file 12864_2018_5368_MOESM1_ESM.pdf]

**a**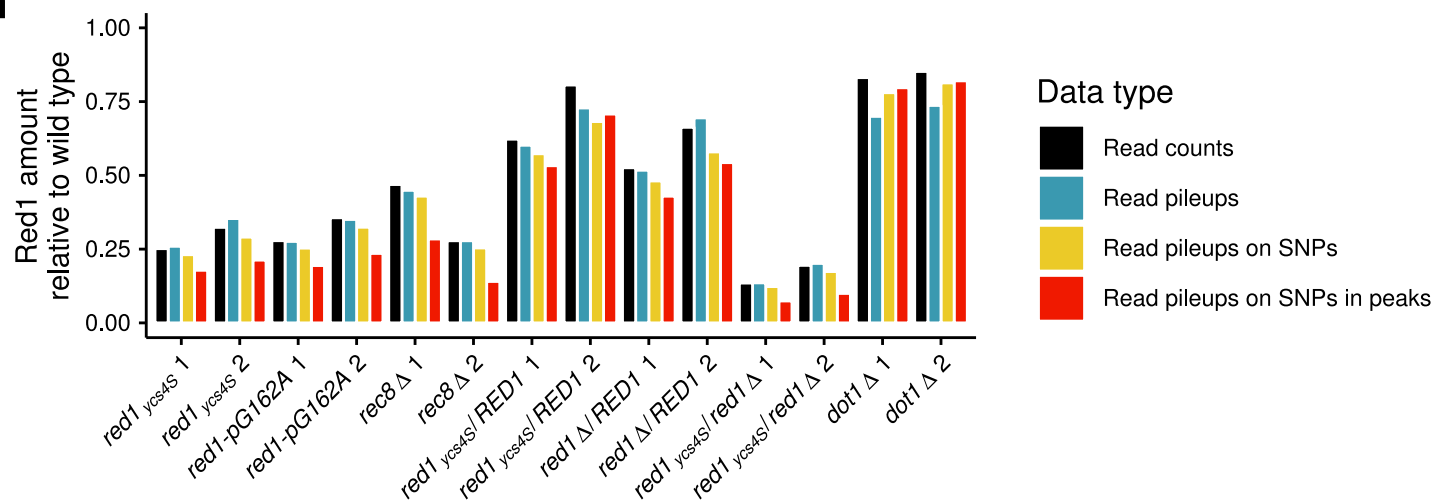**b**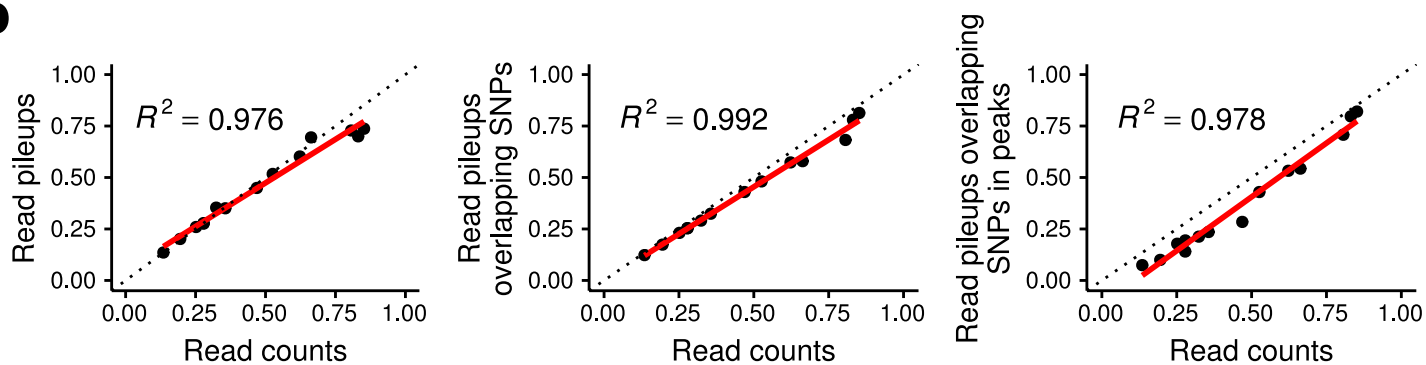

Supplement: Supplementary file 2 — Figure S2. Spike-in normalization factors calculated using different input data types. (PDF 136 kb) [file 12864_2018_5368_MOESM2_ESM.pdf]

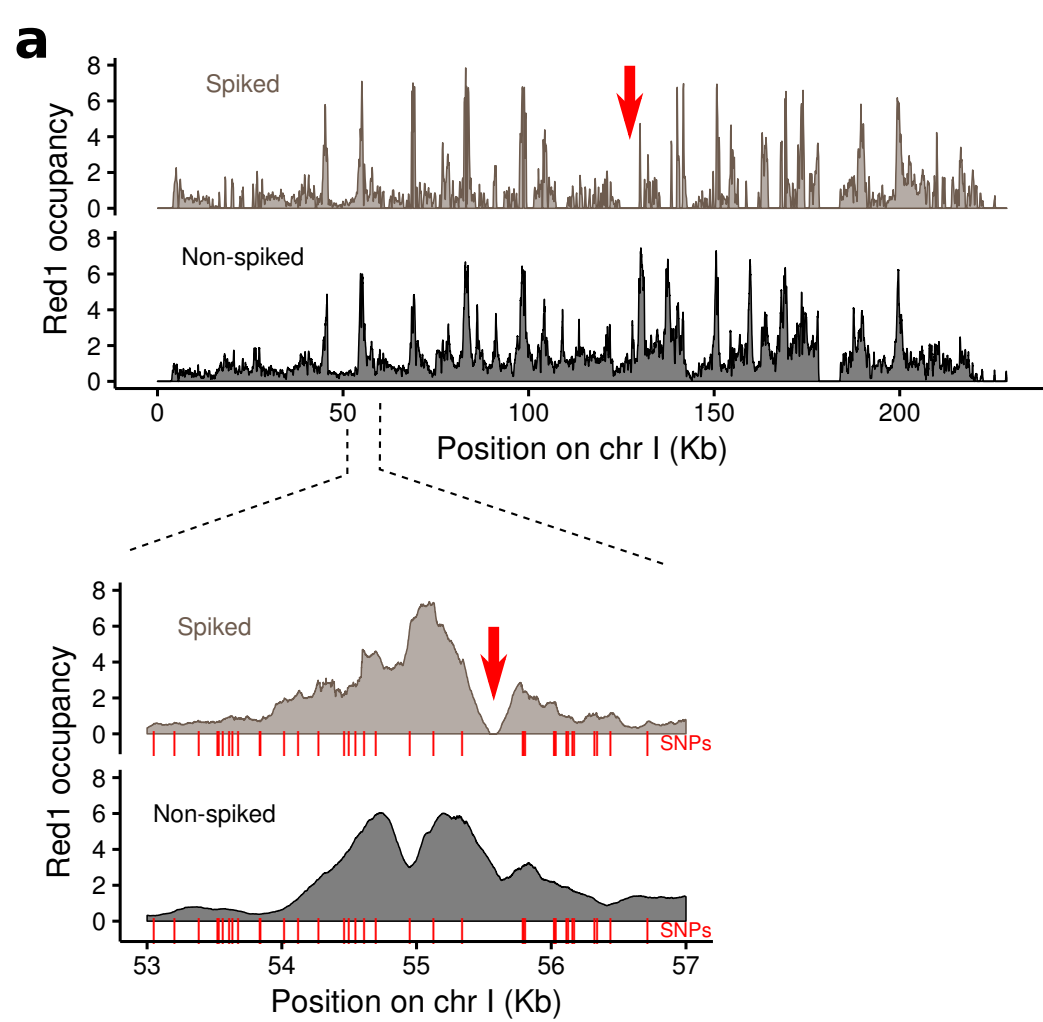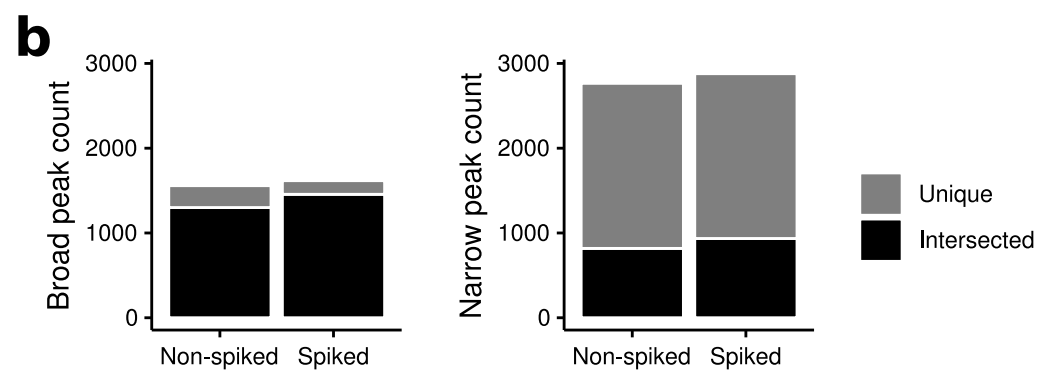

Supplement: Supplementary file 3 — Figure S3. Same-species spike-in causes loss of information but does not affect the overall target distribution patterns. (PDF 440 kb) [file 12864_2018_5368_MOESM3_ESM.pdf]

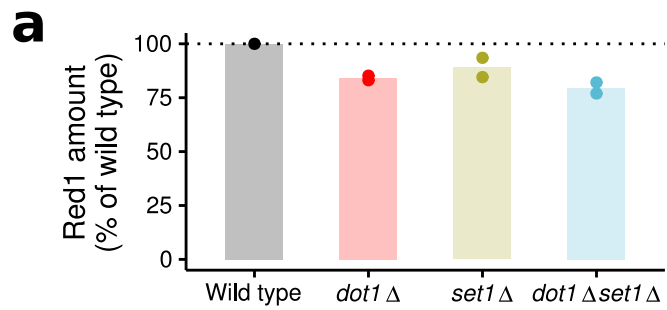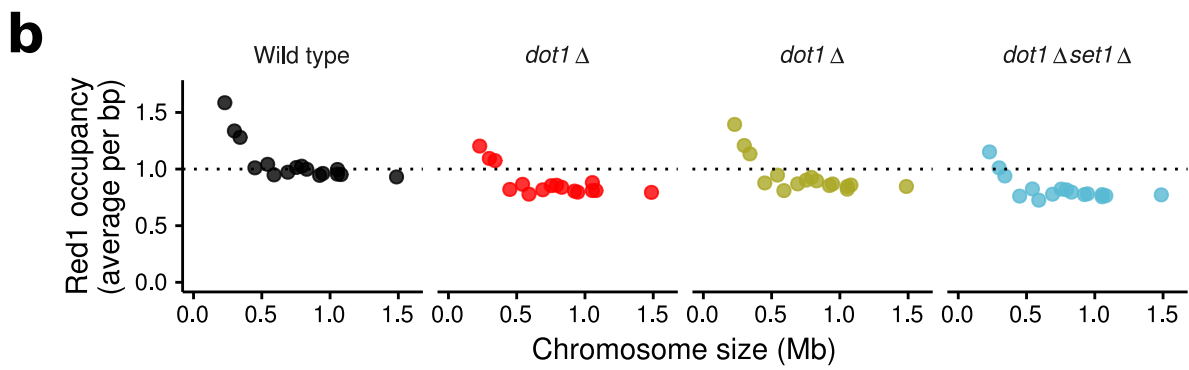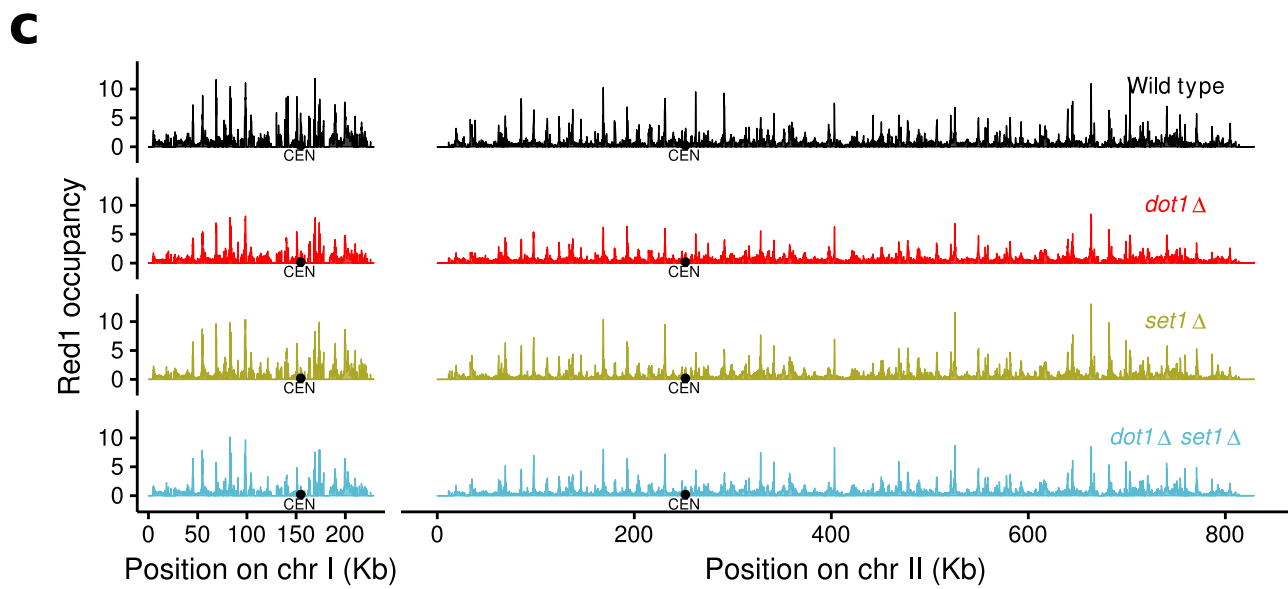

Supplement: Supplementary file 4 — Figure S4. Red1 occupancy is mildly decreased in histone methyltransferase mutants relative to wild type. (PDF 280 kb) [file 12864_2018_5368_MOESM4_ESM.pdf]
