# Supplementary material for: SNP-ChIP: a versatile and tag-free method to quantify changes in protein binding across the genome
Source: BMC Genomics. 2019 Jan 17;20:54. doi: 10.1186/s12864-018-5368-4 (PMC6337847; doi:10.1186/s12864-018-5368-4)
Supplement: Supplementary file 5 — Supplementary Material Contains legends for Figures S1 – S4 and Table S1 (Strains used in this study).. (DOCX 30.0 kb) [file 12864_2018_5368_MOESM5_ESM.docx]

Supplementary Material

SNP-ChIP: A versatile and tag-free method to quantify changes in protein binding across the genome

Luis A. Vale-Silva, Tovah E. Markowitz, and Andreas Hochwagen

Legends for Figures S1 – S4

Table S1

Supplementary References

**Supplementary Figures**

**Figure S1.** Distribution of distances between consecutive single-nucleotide polymorphisms (SNPs) found between the SK1 and S288c yeast genomes. (a) Whole-genome data. (b) Data for individual chromosomes (excluding outlier regions with a combined length of ~10 Kbps on chromosome I showing very high SNP density). Values above 500 bp are not displayed. We note that the median is the more informative statistic in this case, since the mean is inflated by inaccurate outliers. These outliers consist, for example, of regions of low-quality reference genome sequence (such as subtelomeric regions) for which many SNPs are not known, and recorded distances between consecutive SNPs can amount to values as high as several Kbps.

**Figure S2.** Spike-in normalization factors calculated using different input data types. (a) Red1 amount relative to wild type for a collection of samples analyzed in this study (*rec8* - Figure 3a, *red1_ycs4_*/*RED1*, *red1Δ*/*RED1*, *red1_ycs4_*/*red1Δ* - Figure 4, *dot1* - Figure S4; see Supplementary Table 1 for a description of the strains). Values are the ratio between test sample and wild type's spike-in normalization factors calculated using one of four different kinds of data: aligned read counts (read counts; standard procedure) or the mean value of the aligned read pileup score at all genomic positions (read pileups), at SNP positions only (read pileups on SNPs), or at SNP positions falling within called signal peaks (read pileups on SNPs in peaks). (b) Comparison of spike-in normalization factors calculated using total read counts with the three other methods for the same samples plotted in (a).

**Figure S3.** Same-species spike-in causes loss of information but does not affect the overall target distribution patterns. (a) Red1 occupancy obtained using a same-species spike-in compared to a non-spiked replicate. The bottom panel shows a zoom-in on a smaller region, with SNPs annotated as vertical red bars. The red arrows point to example regions where signal is lost in the spiked sample due to the lack of SNPs. (b) Comparison of narrow and broad peaks produced using MACS2 to highlight the proportion of peaks that overlap between non-spiked and spiked samples (intersected) or are unique to each of the samples.

**Figure S4.** Red1 occupancy is mildly decreased in histone methyltransferase mutants relative to wild type. (a) Target protein Red1 levels relative to wild type produced by SNP-ChIP. Points represent individual replicate values and bars represent average value. (b) Spike-in-normalized average Red1 signal on individual chromosomes. (c) Spike-in-normalized fragment pileup produced using MACS2 with SPMR (fragment pileup per million reads) sequencing depth normalization plotted on two example chromosomes.

**Table S1.** Strains used in this study.

| **Strain name** | **Genotype** | **Back**  **ground** | **Reference** | **Figures** |
| --- | --- | --- | --- | --- |
| NKY1551 | *MATa/MATα, ho::LYS2/”, lys2/”, ura3/”, leu2::hisG/”, his4B::LEU2/his4X::LEU2(Bam)-URA3, arg4-BglII/arg4-Nsp* | SK1 | [^1^](#_ENREF_1) | All figures |
| H7011 | *MATa/MATα, ho::LYS2/”, lys2/”, ura3/URA3, leu2::hisG/LEU2, his3::hisG/HIS3, trp1::hisG/TRP1, red1_ycs4S_/red1_ycs4S_* | SK1 | [^2^](#_ENREF_2) | 1, 4, S2 |
| H8218 | *MATa/MATα, ho::LYS2/”, lys2/”, ura3/URA3, leu2::hisG/LEU2, his3::hisG/HIS3, trp1::hisG/TRP1, red1_ycs4S_*/*RED1* | SK1 | [^2^](#_ENREF_2) | 1, 4, S2 |
| H8219 | *MATa/MATα, ho::LYS2/”, lys2/”, ura3/URA3, leu2::hisG/LEU2, his3::hisG/HIS3, trp1::hisG/TRP1, red1_ycs4S_*/*red1Δ::KanMX4* | SK1 | [^2^](#_ENREF_2) | 1, 4, S2 |
| H8220 | *MATa/MATα, ho::LYS2/”, lys2/”, ura3/URA3, leu2::hisG/LEU2, his3::hisG/HIS3, trp1::hisG/TRP1, red1Δ::KanMX4/RED1* | SK1 | [^2^](#_ENREF_2) | 1, 4, S2 |
| H9048 | *MATa/MATα, ho::LYS2/”, lys2/”, ura3/URA3, leu2::hisG/LEU2, his3::hisG/HIS3, trp1::hisG/TRP1, HphMX4::red1-pG162A/HphMX4::red1-pG162A* | SK1 | [^2^](#_ENREF_2) | 2, S2, S3 |
| H4206 | *MATa/MATα, ho::LYS2/”, lys2/”, ura3/”, leu2::hisG/”, his4B::LEU2/his4X::LEU2(Bam)-URA3, arg4-BglII/arg4-Nsp*  *spo11-Y135F-HA::URA3/spo11-Y135F-HA::URA3* | SK1 | This study | 4 |
| H8104 | *MATa/MATα, ho::LYS2/”, lys2/”, ura3/URA3, leu2::hisG/LEU2, his3::hisG/HIS3, trp1::hisG/ trp1::hisG, dot1Δ::TRP1/dot1Δ::TRP1* | SK1 | This study | S2, S4 |
| H8151 | *MATa/MATα, ho::LYS2/”, lys2/”, ura3/”, leu2::hisG/”, his4B::LEU2/his4X::LEU2(Bam)-URA3, arg4-BglII/arg4-Nsp rec8::HIS3/rec8::HIS3* | SK1 | This study | 3, S2 |
| H8583 | *MATa/MATα, ho::LYS2/”, lys2/”, ura3/URA3, leu2::hisG/LEU2, trp1::hisG/”, dot1Δ::TRP1/dot1Δ::TRP1*  *set1Δ::KanMX6/set1Δ::KanMX6* | SK1 | This study | S4 |
| H8584 | *MATa/MATα, ho::LYS2/”, lys2/”, ura3/URA3, leu2::hisG/LEU2,*  *set1Δ::KanMX6/set1Δ::KanMX6* | SK1 | This study | S4 |
| H9120 | *MATa/MATα, ho::LYS2/”, lys2/”, ura3/URA3, leu2::hisG/LEU2, his3::hisG/HIS3, trp1::hisG/”, hop1::LEU2/hop1::LEU2* | SK1 | This study | 3 |
| H8644 | *MATa/MATα, his3Δ1/his3Δ1::pRS303::HIS3, leu2Δ0/leu2Δ::pRS305::LEU2,*  *lys2Δ0/ lys2Δ::pRS307::LYS2, ura3Δ0/URA3(SK1),*  *RME1(ins-308a)/RME1(ins-308a), TAO3(E1493Q)/TAO3(E1493Q), MKT1(D30G)/MKT1(D30G)* | S288c | This study | All figures |

**Supplementary References**

1 Bishop, D. K., Park, D., Xu, L. & Kleckner, N. *DMC1*: a meiosis-specific yeast homolog of *E. coli* recA required for recombination, synaptonemal complex formation, and cell cycle progression. *Cell* 69, 439-456 (1992).

2 Markowitz, T. E. *et al.* Reduced dosage of the chromosome axis factor Red1 selectively disrupts the meiotic recombination checkpoint in *Saccharomyces cerevisiae*. *PLoS Genet* 13, e1006928, doi:10.1371/journal.pgen.1006928 (2017).
